# Neutrophil-to-Lymphocyte Ratio as a Prognostic Biomarker in Pancreatic Neuroendocrine Neoplasms

**DOI:** 10.3390/cancers18213427

**Published:** 2026-10-22

**Authors:** Beata Jabłońska

**Affiliations:** Department of Digestive Tract Surgery, Medical University of Silesia, Medyków 14 Street, 40-752 Katowice, Poland; bjablonska@poczta.onet.pl

**Keywords:** pancreatic neuroendocrine neoplasms, PNEN, neutrophil-to-lymphocyte ratio, NLR, tumor microenvironment, prognostic biomarker, systemic inflammation

## Abstract

Background: Pancreatic neuroendocrine neoplasms (PNENs) represent a rare, heterogeneous group of tumors with highly variable clinical outcomes. While standard clinicopathological parameters like World Health Organization (WHO) grading and TNM staging provide critical prognostic data, they do not fully explain the survival disparities observed in patients with similar tumor characteristics. This narrative review evaluates the clinical and prognostic significance of the neutrophil-to-lymphocyte ratio (NLR)—a widely accessible marker of systemic inflammation—in patients with PNENs. Methods: A comprehensive literature review was conducted to synthesize current evidence regarding the prognostic utility of peripheral NLR levels. Results: Studies conducted specifically in pancreatic neuroendocrine tumors (PNETs) suggest that elevated peripheral blood NLR is associated with adverse clinicopathological features and, in several cohorts, with shorter overall survival (OS) and recurrence-free survival (RFS). However, the strength and independence of these associations vary across studies. Evidence from mixed gastroenteropancreatic neuroendocrine neoplasm (GEP-NEN) cohorts undergoing systemic treatment or peptide receptor radionuclide therapy (PRRT) provides complementary insights into the predictive capacity of NLR regarding treatment response, though it remains indirect evidence for PNETs, particularly when pancreatic-specific subgroup results are unavailable. Differences in tumor differentiation, grade, stage, treatment setting, and NLR cut-off values limit direct comparisons across studies. Conclusions: NLR is an accessible, candidate adjunctive prognostic biomarker in PNETs, particularly in surgically treated populations. However, the evidence remains highly heterogeneous, and its clinical utility and standardized thresholds remain unvalidated. Proposed applications in treatment selection or surveillance intensity should be strictly regarded as investigational and hypothesis-generating, rather than validated clinical strategies. NLR should complement, rather than replace, established clinicopathological prognostic factors.

## Simple Summary

Pancreatic neuroendocrine neoplasms (PNENs) are rare and unpredictable tumors. Standard medical tools, such as tumor staging and grading, help predict patient outcomes, but they are not always perfectly accurate for all patients. Recent studies show that the systemic inflammatory response plays a crucial role in cancer growth and metastasis. The neutrophil-to-lymphocyte ratio (NLR) is a simple score calculated from a standard, routine blood test. It measures the balance between white blood cells that can inadvertently help the tumor grow (neutrophils) and cells that fight the disease (lymphocytes). This review collects and explains current research regarding the use of NLR in patients with PNENs. Ultimately, NLR is a low-cost, widely available biomarker that may serve as a potential adjunctive tool to help clinicians better understand a patient’s risk profile alongside established clinical factors. However, due to significant variations in testing thresholds and potential confounding factors, its routine clinical utility and ability to guide personalized treatment plans remain unvalidated. Future standardized and prospective trials are necessary before it can be formally implemented in clinical practice.

## 1. Introduction

Neuroendocrine neoplasms (NENs) comprise a heterogeneous group of tumors derived from widely distributed neuroendocrine cells, most commonly arising from the gastroenteropancreatic and bronchopulmonary tracts. NENs are divided into neuroendocrine tumors (NETs) and the more aggressive neuroendocrine carcinomas (NECs) according to proliferation index and differentiation. Gastroenteropancreatic NETs (GEP-NETs) include carcinoid tumors of the gastrointestinal tract and pancreatic NETs (PNETs) [1,2,3,4]. These tumors are divided based on their embryological origin into three groups: those originating from the foregut (thymus, esophagus, lung, stomach, duodenum, pancreas), midgut (appendix, ileum, cecum, ascending colon), and hindgut (distal large bowel, rectum) [5].

Pancreatic NENs (PNENs), including pancreatic NETs (PNETs) and pancreatic NECs (PNECs), are a rare and heterogeneous subgroup of neoplasms originating from the endocrine cells of the pancreas. Although they represent only a small part of pancreatic neoplasms, their incidence has increased recently due to advances in imaging techniques, endoscopic procedures, and pathological diagnostics. In contrast to pancreatic ductal adenocarcinoma (PDAC), PNENs display a broad spectrum of biological behavior, with clinical outcomes ranging from prolonged survival in patients with localized disease to rapid progression and poor survival in patients with advanced or metastatic neoplasms. The first case of a tumor originating from the pancreatic endocrine cells was described by Nicholls in 1902 [1,2,3,4,5,6,7].

PNETs are generally slow-growing tumors, having different overall survival (OS) depending on the tumor grading and staging [2,7]. In the study by Sonbol et al. [2], for the entire cohort, the median OS (mOS) was 68 months (95% CI [64, 73]). The 5-year OS rates were 83.19% (standard error (SE) = 0.86%) for localized, 67.36% (SE = 1.31%) for regional, and 28.13% (SE = 0.79%) for metastatic disease. When stratified by tumor differentiation, 5-year OS rates reached 77.33% (SE = 0.87%), 63.06% (SE = 1.92%), and 20.04% (SE = 1.60%) for grades I, II, and III, respectively. Combined analysis demonstrated 5-year OS rates of 87.28%, 79.69%, and 46.24% for localized grades I–III; 80.99%, 72.49%, and 38.19% for regional grades I–III; and 51.03%, 45.16%, and 12.37% for metastatic grades I–III. The study by Franco et al. [7] reported 33% 5-year overall survival rates at 5 years in non-functional pancreatic neuroendocrine carcinomas [7]. 

They are divided into non-functioning and functioning tumors. Recently, non-functioning PNETs are increasing and account for >60% of all pancreatic neuroendocrine neoplasms [1,2]. Prognosis in PNETs is primarily determined by established clinicopathological factors, including tumor differentiation, World Health Organization (WHO) grade, Ki-67 proliferation index, mitotic count, as well as Tumor Node Metastasis (TNM) staging involving lymph node (LN) invasion, and the presence of distant metastases. Despite the important prognostic value of the above-mentioned parameters, they do not fully explain the variability in clinical outcomes observed in patients with apparently similar tumor characteristics. This has prompted the search for additional biomarkers that may improve risk stratification and support clinical decision-making [1,3,8].

Biomarkers reflecting systemic inflammation are important prognostic factors in numerous neoplasms. It is known that persistent inflammation contributes to multiple stages of tumor development by promoting cell proliferation, angiogenesis, immune evasion, and metastatic spread in neoplasms. These processes are mediated through complex interactions between tumor cells and components of the tumor microenvironment (TME), including inflammatory cells, fibroblasts, endothelial cells, and a wide range of cytokines and growth factors such as interleukin-6, tumor necrosis factor-α, transforming growth factor-β, and vascular endothelial growth factor [9,10,11,12].

The neutrophil-to-lymphocyte ratio (NLR), calculated from a standard complete blood count using the proportion of absolute neutrophil count in circulating blood to absolute lymphocyte count, has emerged as one of the most extensively studied inflammation-based biomarkers in oncology. Neutrophils can facilitate tumor progression through the secretion of proinflammatory cytokines, angiogenic mediators, matrix-remodeling enzymes, and neutrophil extracellular traps, whereas lymphocytes play a central role in antitumor immune surveillance. Consequently, an elevated NLR may reflect both an enhanced systemic inflammatory response and impaired host immunity, providing a plausible biological explanation for its association with unfavorable clinical outcomes [13,14,15,16,17,18].

The aim of this narrative review is to provide a comprehensive overview of the current evidence regarding the prognostic significance of NLR in pancreatic neuroendocrine neoplasms.

## 2. The WHO Classification of Pancreatic Neuroendocrine Neoplasms (PNENs)

**Figure 1:**
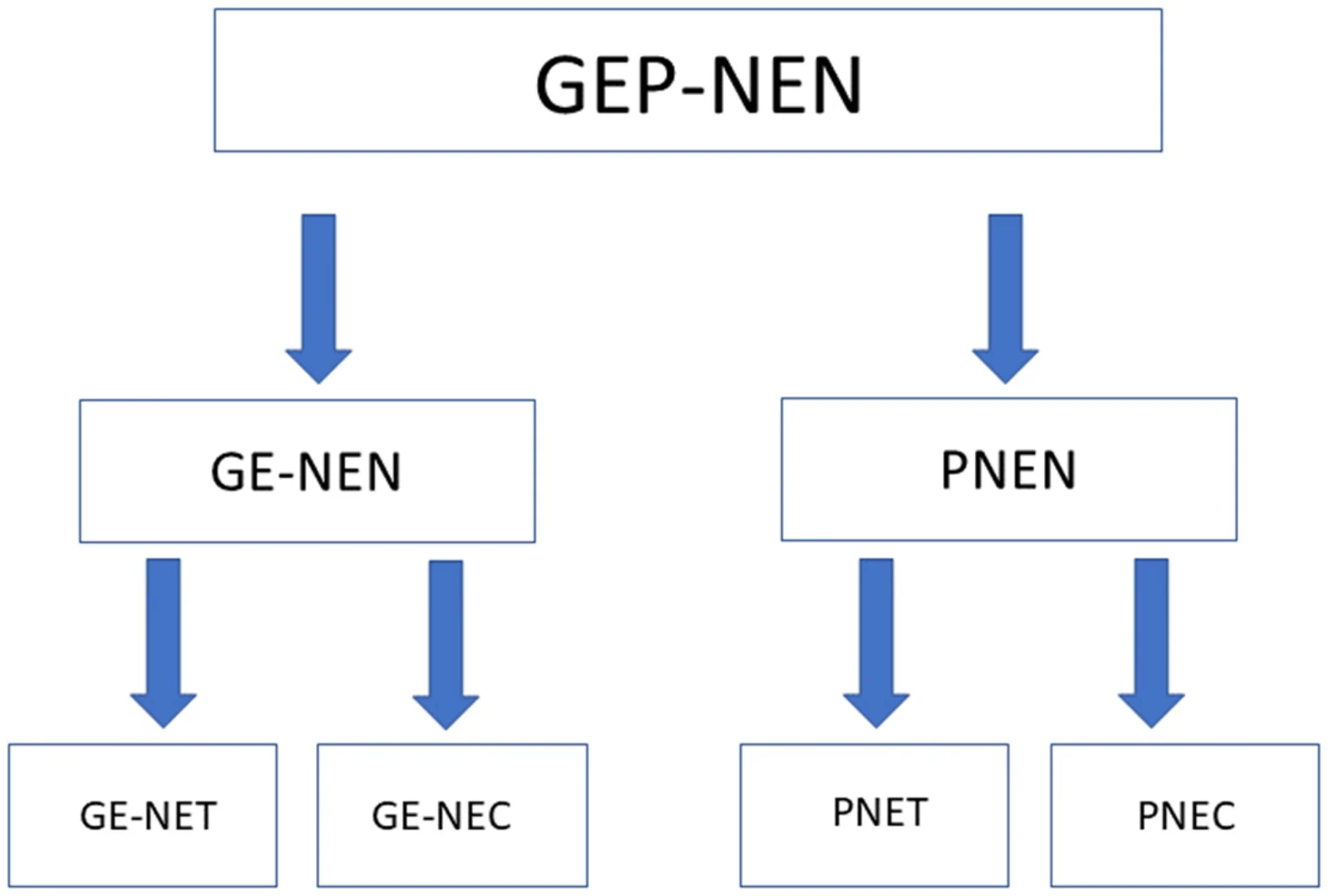
**Classification of GEP-NENs. Abbreviations:** GEP-NENs, gastroenteric-pancreatic neuroendocrine neoplasm; GE-NEN, gastroenteric neuroendocrine neoplasm; GE-NETs, gastroenteric neuroendocrine tumor; GE-NECs, gastroenteric neuroendocrine carcinoma; PNEN, pancreatic neuroendocrine neoplasm; PNET, pancreatic neuroendocrine tumor; PNEC, pancreatic neuroendocrine carcinoma.

As mentioned above, PNENs are divided into PNETs and PNECs. The classification of GEP-NENs is shown in Figure 1 [1,2,3,4]. According to the 2022 WHO (5th edition) classification, PNETs are divided into G1, G2, and G3 by the number of mitoses and the Ki-67 proliferation index (Table 1) [1,2]. 

## 3. Biological Rationale: TME in PNENs

Tumor-associated inflammation is increasingly recognized as an important component of the TME and may contribute to cancer progression through cytokine and chemokine signaling, immune dysregulation, angiogenesis, extracellular-matrix remodeling, and immune evasion. In PNENs, the TME comprises multiple immune and stromal cell populations whose composition varies according to tumor differentiation, grading, and staging [9,10,11,12,13,14,15,16,17,18,19,20,21]. 

In particular, studies of PNEN tissue have demonstrated associations between immune-cell infiltration and tumor aggressiveness. These observations support a biological association between the TME and PNEN behavior. Neutrophils may promote tumor progression through several mechanisms, including the release of proangiogenic mediators, matrix metalloproteinases, reactive oxygen species, and neutrophil extracellular traps, thereby facilitating angiogenesis, extracellular-matrix remodeling, invasion, and immune suppression [3,22,23]. Conversely, lymphocytes, particularly cytotoxic T cells, are central components of antitumor immunity, and their presence within the TME may reflect an effective host immune response [21,24]. 

Recent studies of PNETs have demonstrated the different composition of the TME according to disease stage and metastatic status. In a cohort of non-functioning PNETs (NF-PNETs), analyzed by Zhang et al. [21], increased tumor-infiltrating neutrophils (TINs) and tumor-associated macrophages (TAMs) were associated with higher tumor grade and poorer RFS and OS, whereas greater intratumoral CD8+ T-cell infiltration was related to prolonged RFS. Importantly, intratumoral TINs remained an independent prognostic factor for RFS and OS after adjustment for WHO grading and TNM staging [21]. In another study by Dieben et al. [25], transcriptomic deconvolution of 83 primary tumors and 30 liver metastases demonstrated that neutrophils were significantly enriched in metastatic lesions compared to primary site tumors that suggest the possible link between liver metastasis, complement pathway activation, and neutrophils infiltration in well-differentiated pNETs [25]. In contrast, Greenberg et al. [24] demonstrated increased numbers of tumor-infiltrating T cells in metastatic PNETs compared to localized tumors. In addition, RNA-Seq analysis indicated that the primary tumors of metastatic PNETs showed significant activation of inflammatory and immune-related pathways [24]. The above-presented contradictory findings indicate that PNET progression is accompanied by substantial remodeling of the immune microenvironment, involving both innate and adaptive immune components. Importantly, the relationship between immune cell infiltration and tumor progression appears complex, as increased immune infiltration may reflect either effective antitumor surveillance or a tumor-promoting inflammatory response depending on the cellular composition and functional state of the infiltrating immune population [21,24,25,26]. Moreover, Immune cells constitute an important component of the PNEN TME. The above-described studies [20,21,23,24,25] suggest that the balance between potentially tumor-promoting innate immune cells and antitumor adaptive immune cells may contribute to the biological heterogeneity of PNENs.

Thus, the TME of PNENs comprises a complex network of immune, stromal, endothelial, and other non-neoplastic cells that interact dynamically with tumor cells and may influence tumor progression, metastatic potential, and response to therapy [20,23,24,25]. Additionally, other components of the innate and adaptive immune systems, such as myeloid-derived suppressor cells (MDSCs), natural killer (NK) cells, B cells, and mastocytes, alongside perineural signaling networks, further contribute to this intricate immunosuppressive and regulatory architecture [19,23,24]. Although the TME of PDAC has been extensively characterized, the immune and stromal landscape of PNENs remains less well understood. Recent studies have demonstrated that the PNEN TME is biologically heterogeneous and different according to tumor grading, disease progression, and metastatic dissemination [21,25].

Beyond immune cells, endothelial cells, fibroblasts, extracellular-matrix components, and vascular signaling pathways may contribute to the characteristic biology of PNENs. Neuroendocrine tumors are highly vascularized, and interactions between tumor cells and endothelial cells are closely related to angiogenesis and tumor growth. Stromal components may additionally modulate immune-cell recruitment, extracellular-matrix remodeling, and the availability of growth factors. According to recent literature, the PNEN TME should be considered an integrated ecosystem rather than a collection of independent cellular populations [19]. Within this ecosystem, the extracellular matrix (ECM) serves as a dynamic, non-cellular structural network that undergoes profound remodeling driven by signaling events between tumor cells and the stroma [19]. In PNENs, alterations in the composition and physical properties of the ECM—such as fiber alignment, density, and elasticity—do not merely provide structural scaffolding but actively direct cell migration, biochemical signaling, and tissue remodeling [19]. This continuous structural modification contributes to a supportive niche that facilitates tumor progression, limits drug delivery, and shapes the local immune landscape, highlighting the critical role of ECM remodeling in defining the aggressive behavior of specific PNEN phenotypes [19].

The biological TME complexity provides a rationale for investigating systemic inflammatory biomarkers as accessible surrogates of tumor–host interactions. Peripheral blood indices such as the neutrophil-to-lymphocyte ratio (NLR), platelet-to-lymphocyte ratio (PLR), and lymphocyte-to-monocyte ratio (LMR) do not directly measure the intratumoral immune landscape, but may reflect systemic consequences of tumor-associated inflammation and immune dysregulation. The association between increased tumor-associated neutrophils and poor outcomes in PNENs, together with the prognostic relevance of circulating NLR in clinical cohorts, provides a potential biological link between tissue-level inflammation and peripheral blood inflammatory indices. However, the imperfect correlation between systemic and intratumoral immune profiles should be recognized when interpreting these biomarkers [21,25].

Overall, current evidence supports a model in which PNEN progression is accompanied by dynamic TME remodeling involving both innate and adaptive immune components. The prognostic significance of this remodeling appears to depend on the cellular composition and functional phenotype of the infiltrate rather than on the overall degree of immune-cell infiltration. Future studies integrating tissue-based immune profiling, spatial transcriptomics, circulating inflammatory markers, and clinicopathological variables may help determine whether systemic inflammatory indices can serve as clinically useful surrogates of specific TME phenotypes in PNENs [20,21,22,23,24,25,26].

**Figure 2:**
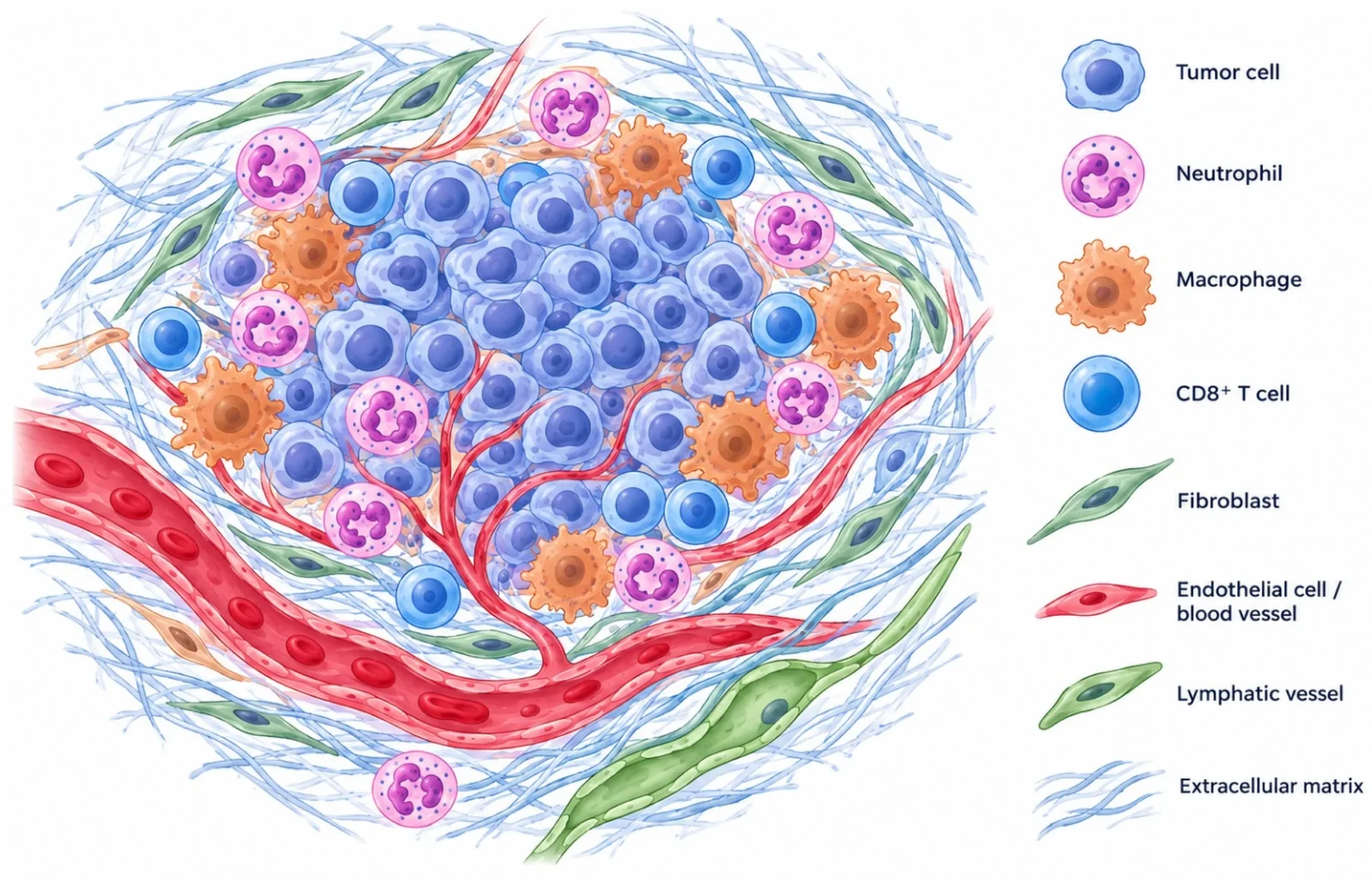
**The tumor microenvironment (TME) of pancreatic neuroendocrine neoplasms (PNENs).**

Given the above-mentioned close relationship between the TME and systemic host–tumor inflammation, peripheral blood-derived inflammatory biomarkers have attracted increasing attention as readily available prognostic indicators in PNENs. Figure 2 summarizes the above-mentioned mechanisms and associations within the TME of PNENs.

## 4. Literature Search Strategy and Study Selection

This narrative review synthesizes and critically evaluates the literature on the associations between the NLR and clinicopathological characteristics, surgical outcomes, and oncological outcomes in PNENs. A non-systematic literature search was conducted in PubMed, with the final search performed on 31 August 2026. The search strategy combined terms for neuroendocrine neoplasms with terms related to NLR: (“Neuroendocrine Tumors” OR “Neuroendocrine Tumor” OR “Tumor, Neuroendocrine” OR “Tumors, Neuroendocrine”) AND (“neutrophil lymphocyte ratio” OR “neutrophil to lymphocyte ratio” OR “neutrophil-to-lymphocyte ratio” OR “neutrophil/lymphocyte ratio” OR “NLR”). The search covered PubMed records from database inception to the final search date.

Eligible publications were full-text articles in English involving adult patients and investigating the prognostic value of NLR regarding clinicopathological characteristics and long-term oncological outcomes, as well as its potential predictive value in forecasting response to specific therapeutic modalities, either as the sole population or as part of a broader GEP-NENs cohort. Retrospective observational studies, systematic reviews, and meta-analyses were considered. Animal and laboratory studies, studies involving pediatric populations, case reports, conference abstracts, non-English-language publications, nonsystematic reviews, and studies outside the scope of this review were excluded. Titles and abstracts were initially assessed for relevance, followed by full-text assessment of potentially eligible publications against the predefined eligibility criteria. 

No relevant publications meeting the review’s eligibility criteria were identified before 2016 or between 1 January and 31 August 2026. Accordingly, the relevant evidence discussed in this review consists of studies published between 2016 and 2025. The included studies are discussed in the following sections.

Although this review focuses on PNENs, eligibility was not restricted to studies involving pancreatic primaries exclusively. Studies of mixed GEP-NEN populations were considered when they reported findings relevant to NLR and clinical outcomes, including pancreatic subgroup analyses where available. To avoid overgeneralization, the evidence was interpreted according to the population studied: (1) studies restricted to PNETs; (2) studies of PNENs in which the proportions of well-differentiated NETs and poorly differentiated PNECs were mixed or incompletely reported; and (3) studies involving multiple primary sites or broader NEN populations. Findings from the latter two categories were considered indirect evidence for PNETs unless a separate pancreatic-specific estimate was reported. Study design, differentiation, grade, stage, and treatment setting were considered when interpreting and comparing findings across studies.

## 5. Literature Review

### 5.1. NLR and Tumor Aggressiveness: Tumor Grade, Ki-67, Tumor Size, and Stage

A consistent theme across the available literature is the association between an elevated NLR and clinicopathological features of more aggressive PNET biology. Beyond these peripheral blood indices, evaluating other potential prognostic biomarkers based on the analysis of good-quality tissue samples remains fundamental, as large-scale aggregate data demonstrate that baseline sample characterization is essential for establishing reliable clinical and pathological risk models. EUS-guided tissue acquisition also has a fundamental role in PanNETs because, in addition to diagnosis, it also provides mitotic rate and Ki-67 index, which define grading [27]. Higher NLR values have repeatedly been observed in tumors with higher WHO grade, increased proliferative activity, larger tumor size, and more advanced TNM stage [28,29,30,31,32,33,34,35,36,37,38,39,40,41,42,43,44,45,46,47,48].

In the study by Arima et al. [29], the mean preoperative NLR increased progressively with tumor grade, from 1.76 ± 0.52 in G1 tumors to 2.23 ± 0.66 in G2 tumors and 3.52 ± 0.67 in G3 tumors. Luo et al. [30] similarly found that an NLR > 2.4 was significantly associated with larger tumor size (*p* = 0.001), TNM stage III–IV (*p* = 0.019), and higher tumor grade (*p* = 0.003). Tong et al. [31] also reported significantly higher NLR values in G3 tumors than in G1–G2 tumors (*p* = 0.015) and in patients with advanced T stage (T3–T4) compared with earlier-stage disease (*p* = 0.044).

The association between NLR and proliferative and pathological features was further supported by Harimoto et al. [37]. Using an NLR cut-off of 3.41, identified by time-dependent ROC analysis (AUC = 0.795, *p* = 0.015), the authors found that high NLR was associated with a higher Ki-67 index, higher mitotic count, higher tumor grade, increased incidence of lymph node metastasis, and higher rates of lymphatic and neural invasion. Similarly, in the large cohort of 620 patients studied by Panni et al. [36], elevated NLR was associated with adverse outcomes alongside established factors including tumor size, Ki-67 category and activity, and tumor grade. Taken together, these findings indicate that elevated NLR generally accompanies a phenotype characterized by increased proliferative activity and more advanced disease.

This relationship was further confirmed by Dikmen et al. [41], who compared 44 patients with PNETs with 44 age- and sex-matched healthy controls. The median NLR was higher in patients with PNETs than in controls (2.4 vs. 1.8), and both NLR and PLR increased progressively with advancing European Neuroendocrine Tumor Society (ENETS) and TNM stage, reaching their highest values in stage IV disease (*p* < 0.05). Similarly, Yang et al. [48], in a cohort of 174 surgically treated patients, found that high NLR was associated with tumors > 2 cm (*p* = 0.001), G2/G3 histology (*p* = 0.015), and more advanced TNM stage (*p* < 0.001). 

The association between NLR and tumor grade is also supported by meta-analytic evidence. Oh et al. [45], analyzing 9 studies, reported that the estimated prevalence of high NLR increased from 15.5% in G1 tumors to 41.9% in the combined G2/G3 group. When analyzed separately, the estimated prevalence was 36.7% for G2 and 57.3% for G3 tumors. Mean NLR values also increased progressively with tumor grade, from 1.912 in G1 tumors to 2.258 in G2 and 3.363 in G3 tumors [45]. 

### 5.2. NLR, Lymph Node Involvement, and Distant Metastasis

The relationship between NLR and metastatic spread has also been investigated, although the evidence is less uniform than that concerning tumor grade. Several studies demonstrated an association between elevated NLR and lymph-node metastasis. Tong et al. [31] identified an NLR cut-off of 2.056 for predicting lymph-node metastasis. In multivariate analysis, NLR was independently associated with lymph-node involvement (HR 6.740, *p* = 0.023), together with T stage (HR 11.940, *p* = 0.005) and tumor grade (HR 10.378, *p* = 0.022).

In contrast, Arima et al. [29] found no association between elevated NLR and lymph-node metastasis. However, NLR ≥ 2.4 was independently associated with the development of postoperative liver metastases (HR 7.570, 95% CI 2.17–30.2; *p* = 0.0016). This discrepancy suggests that the relationship between systemic inflammation and metastatic dissemination may differ according to the site and timing of metastasis.

Other studies support a broader association between inflammatory indices and metastatic disease. Salman et al. [28] reported significantly higher NLR and PLR values in patients with advanced TNM stage and metastatic disease (*p* = 0.0001). In the prospective study by Gaitanidis et al. [32], distant metastasis and NLR > 2.3 were both independently associated with worse progression-free survival (PFS), with HRs of 2.80 (95% CI 1.26–6.21; *p* = 0.012) and 2.53 (95% CI 1.05–6.08; *p* = 0.038), respectively. Median PFS was significantly shorter in patients with NLR > 2.3 than in those with lower NLR values (25.3 vs. 37.9 months; *p* = 0.005).

Composite inflammatory or clinicopathological indices may also reflect metastatic potential. Zhou et al. [38] reported that a high score, including preoperative main pancreatic duct dilation (PD) (diameter more than 3 mm) and the NLR (PD-NLR score), was associated with tumor size, T stage, lymph-node metastases, distant metastases, hormone production, perineural invasion, and WHO classification. Moreover, PD-NLR score remained independently associated with both worse overall survival (OS) (HR 16.159, 95% CI 1.902–137.304; *p* = 0.011) and disease-free survival (DFS) (HR 7.356, 95% CI 1.495–36.194; *p* = 0.014). In addition, patients with a high NLR had significantly shorter OS compared to those with a low NLR (HR 8.837, 95% CI 2.990–26.117, *p* < 0.001).

Dikmen et al. [41] found significantly higher NLR values in patients with lymph-node metastases than in those without nodal involvement (*p* = 0.001), while NLR, PLR, and platelet counts increased with advancing ENETS and TNM stage [41].

However, more recent evidence suggests that NLR may not independently predict nodal involvement in all neuroendocrine tumor populations. Ocakli et al. [49], in a retrospective study of 132 patients with gastroenteropancreatic neuroendocrine neoplasms, found that small-bowel tumor location (*p* = 0.002), grade III tumors (*p* = 0.008), and tumor size ≥ 2 cm (*p* = 0.017) independently predicted pathological lymph-node metastasis, whereas NLR was not predictive (*p* = 0.998) [49]. Thus, although elevated NLR may correlate with nodal disease in some PNET cohorts, its value as an independent predictor of lymph-node metastasis remains uncertain. 

Literature regarding distant metastasis similarly indicates an association with aggressive disease, although NLR may be more informative as a general marker of systemic tumor burden than as a specific predictor of one metastatic pattern. This interpretation is consistent with the heterogeneity observed between surgical cohorts, advanced disease populations, and mixed gastroenteropancreatic NEN cohorts [29,30,31,32,38,41,49,50].

### 5.3. NLR and Hormonal Function

The evidence concerning the relationship between NLR and hormonal function is more limited and inconsistent. Luo et al. [30] found only a borderline association between NLR > 2.4 and functioning status (*p* = 0.084), suggesting that hormonal activity was not clearly related to systemic inflammatory status in that cohort. In contrast, Zhou et al. [38] reported a significant association between PD-NLR score and hormone production (*p* = 0.006). In this study, the optimal cut-off value of NLR was 3.13.

Furthermore, Harimoto et al. [37] identified non-functional PNETs as one of the factors associated with recurrence in univariate analysis, together with elevated NLR, larger tumor size, higher grade, increased Ki-67 index and mitotic count, and lymphovascular or neural invasion. These observations suggest that the prognostic significance of systemic inflammatory markers may differ between functional and non-functional tumors; however, the available evidence remains insufficient to establish a consistent relationship between NLR and hormonal activity.

Miura et al. [43], in a cohort of 120 patients undergoing curative surgery for well-differentiated PNETs, demonstrated that the prognostic significance of NLR differed according to functional status. Using an optimal NLR cut-off of 2.62, elevated preoperative NLR independently predicted recurrence in the overall cohort (HR 3.49, 95% CI 1.05–11.7; *p* = 0.042). However, the association between elevated NLR and shorter RFS was significant in nonfunctional PNENs (HR 4.95, 95% CI 1.430–20.1; *p* = 0.002) but not in functional tumors (*p* = 0.094) [43].

In contrast, Yang et al. [48] found no significant differences in tumor functionality between high- and low-NLR groups. Therefore, current evidence does not support a consistent direct relationship between NLR and hormone production; rather, functionality may modify the prognostic relevance of NLR in selected patient populations [30,38,43,48].

### 5.4. NLR and Pathological Features Associated with Recurrence and Surgical Outcomes

Elevated NLR has also been associated with pathological features linked to recurrence and, in some studies, with adverse surgical characteristics. Arima et al. [29] also identified NLR ≥ 2.4 as an independent predictor of postoperative recurrence (HR 6.012, *p* = 0.0035), together with tumor size ≥ 2 cm (HR 6.760, *p* = 0.0049). Similarly, Tong et al. [31] reported that an NLR > 1.40 was associated with shorter recurrence-free survival (RFS), with mean RFS of 61.1 ± 4.4 months in the high-NLR group compared with 63.8 ± 2.9 months in the low-NLR group (*p* < 0.05). In the cohort studied by Harimoto et al. [37], patients with NLR ≥ 3.41 had higher rates of lymphatic and neural invasion and greater intraoperative blood loss. In multivariate analysis, high NLR, NET G2/G3 grade, and synchronous hepatic resection were identified as independent risk factors for recurrence following curative resection. Patients with high NLR had significantly poorer recurrence-free survival (RFS). Miura et al. [43] found that preoperative NLR > 2.62 independently predicted recurrence following curative surgery (HR 3.49, 95% CI 1.05–11.7; *p* = 0.042) [43].

More recent evidence from Yang et al. [48] provides further support for the prognostic relevance of systemic inflammatory indices after PNET surgery. Patients who subsequently developed tumor progression had significantly higher median NLR (2.923 vs. 2.284, *p* = 0.039) and PLR (174.4 vs. 137.2, *p* = 0.011), whereas LMR was lower (3.733 vs. 4.857, *p* = 0.001). The optimal cut-off values for predicting postoperative progression were 2.28 for NLR, 4.36 for LMR, and 120.91 for PLR. Progression was significantly more frequent in patients with high NLR (35.82% vs. 17.76%, *p* = 0.007), low LMR (37.78% vs. 10.71%, *p* < 0.001), and high PLR (32.26% vs. 16.05%, *p* = 0.013) [48]. Although high NLR was associated with a 2.352-fold increased risk of progression in univariate analysis (HR 2.352, 95% CI 1.286–4.303; *p* = 0.006), only LMR remained independently associated with PFS after multivariable adjustment (HR 3.128, 95% CI 1.107–8.836; *p* = 0.031), together with pathological grade and age [48]. This finding highlights an important limitation of NLR: it may correlate with adverse outcomes without consistently retaining independent prognostic significance after adjustment for other clinicopathological and inflammatory variables. 

Overall, the above-presented findings suggest that elevated NLR may identify patients at increased risk of recurrence after surgical treatment. However, direct evidence supporting NLR as a predictor of standardized postoperative complications remains limited, as most studies focused on recurrence, pathological invasion, intraoperative blood loss, or long-term survival rather than postoperative morbidity.

### 5.5. NLR and Survival Outcomes

The strongest and most consistent evidence concerns the association between elevated NLR and adverse survival outcomes. Salman et al. [28] reported a strong negative correlation between NLR and progression-free survival (PFS) (r = −0.862, *p* < 0.001). Using the median NLR value of 2.17 as a threshold, the ROC analysis predicted a PFS of 11.5 months, with an AUC of 0.94, sensitivity of 98.5%, and specificity of 53.7%. Similarly, Arima et al. [29] found that patients with NLR ≥ 2.4 had significantly shorter OS (*p* = 0.0481) and RFS (*p* < 0.0001) than those with NLR < 2.4. Luo et al. [30] also demonstrated that NLR > 2.4 remained independently associated with worse OS in multivariate analysis (HR 3.60, *p* = 0.011), together with TNM stage III–IV (HR 6.70, *p* = 0.001) and G3 tumor grade (HR 6.31, *p* = 0.004).

The prospective study by Gaitanidis et al. [32] provided additional evidence for the prognostic relevance of NLR (median follow-up of 15 months, range 12–73 months). An NLR > 2.3 was independently associated with shorter PFS (HR 2.53, 95% CI 1.05–6.08; *p* = 0.038). In the same study, distant metastatic disease was also an independent predictor of worse PFS (HR 2.80, 95% CI 1.26–6.21; *p* = 0.012).

Evidence from larger cohorts supports this association. Panni et al. [36], in a study of 620 patients with resectable PNETs, used cut-off values of 3.7 for NLR and 0.5 for monocyte-to-lymphocyte ratio (MLR). NLR ≥ 3.7 was independently associated with worse RFS (HR 1.79, 95% CI 1.20–2.66; *p* = 0.01). Elevated NLR and MLR were also associated with worse OS in univariate analyses, although the contribution of individual inflammatory markers varied after adjustment for established clinicopathological factors.

Harimoto et al. [37] further demonstrated that an NLR ≥ 3.41 was associated with poorer RFS after potentially curative pancreatic resection. Taken together, these studies suggest that elevated preoperative NLR may provide additional prognostic information in surgically treated patients. Oh et al. [45] reported that elevated NLR was associated with significantly poorer OS (HR 2.180, 95% CI 1.499–3.169) and RFS (HR 2.462, 95% CI 1.677–3.615) in patients with PNETs [45].

Worapongpaiboon et al. [51] subsequently analyzed 105 studies involving 20,138 patients with pancreatic malignancies and cystic neoplasms, including PNETs. In the PNET subgroup, high NLR was associated with worse OS in both univariable analysis (HR 4.85, 95% CI 2.05–11.46) and multivariable analysis (HR 2.57, 95% CI 1.50–4.39). Similarly, high NLR was associated with worse RFS in univariable analysis (HR 4.64, 95% CI 2.52–8.51) and multivariable analysis (HR 3.05, 95% CI 1.80–5.16) [51].

A second meta-analysis by Wang et al. [50], including 18 studies and 2995 patients with gastroenteropancreatic neuroendocrine neoplasms, also confirmed the adverse prognostic association of elevated NLR. In the PNEN subgroup, high NLR was associated with poorer OS (HR 3.98, 95% CI 1.92–8.25; *p* = 0.0002) and RFS (HR 2.27, 95% CI 1.63–3.16; *p* < 0.00001). Among surgically treated patients, elevated NLR was associated with worse OS (HR 2.93, 95% CI 2.01–4.28) and RFS (HR 4.29, 95% CI 2.97–6.18), whereas it was not useful for predicting OS in non-operative patients [50]. 

These meta-analytic findings provide stronger support for the prognostic relevance of NLR, particularly in surgically treated PNEN populations. Nevertheless, the magnitude of the effect varies considerably between studies, emphasizing the influence of disease stage, treatment setting, and NLR cut-off selection.

### 5.6. NLR in Systemic Therapy and PRRT

The predictive role of NLR may differ according to treatment setting. Chan et al. [42] pooled data from the phase III RADIANT-3 and RADIANT-4 trials, including 410 and 302 patients, respectively. In the pooled population, higher NLR (≥2.58) was associated with shorter median PFS compared with lower NLR (<2.58) (8.1 vs. 10.8 months; HR 0.75, 95% CI 0.61–0.92; *p* = 0.0064). Lower LMR was also associated with shorter PFS (7.4 vs. 11.1 months; HR 0.70, 95% CI 0.57–0.86; *p* < 0.001), and similar associations were observed in the everolimus and placebo subgroups [42].

In contrast, the results from PRRT-treated populations are less consistent. Ohlendorf et al. [44], in 33 patients with advanced GEP-NETs, found that NLR was higher in non-responders than responders (6.6 ± 3.1 vs. 2.8 ± 1.1), although the difference was not statistically significant (*p* = 0.056). Baseline NLR was significantly associated with changes in whole-body tumor burden after two cycles of PRRT (*p* = 0.004), but was not associated with PFS (HR 0.86, 95% CI 0.29–2.50; *p* = 0.78) [44].

Casas et al. [47], in a multicenter study of 40 patients undergoing PRRT, reported that elevated baseline NLR (the cut-off value used to distinguish normal from pathological index values was 2.61), albumin levels/lymphocyte count (ALR), and derived NLR (d-NLR) (cut-off value 1.71) were associated with significantly shorter PFS (*p* = 0.001, *p* = 0.03, and *p* = 0.001, respectively), whereas MLR and PLR were not significant baseline predictors. None of the inflammatory indices measured after the second PRRT cycle demonstrated predictive value for PFS [47]. These results suggest that pretreatment inflammatory status may act as a baseline predictive marker for radionuclide therapy response, rather than on-treatment measurements, but further validation is required [47].

Beyond systemic therapies and radionuclide protocols, exploring the potential implications of baseline inflammatory markers across different treatment modalities has extended to localized interventions, including ablative therapies. In particular, aggregate data regarding endoscopic ultrasound-guided radiofrequency ablation (EUS-RFA) demonstrate high clinical efficacy and a favorable safety profile for both functional and non-functional tumors, suggesting a critical need to investigate whether circulating surrogates like NLR can systematically improve patient selection and predict response to these emerging minimally invasive ablative modalities [52].

### 5.7. Controversy Regarding the Independent Prognostic Value of NLR

Despite the overall trend toward an association between elevated NLR and unfavorable outcomes, the prognostic value of NLR has not been confirmed consistently across all studies. In the CLARINET study, Grenader et al. [39] found that NLR was not significantly associated with PFS in patients with advanced intestinal and pancreatic NETs, either when evaluated according to NLR tertiles or using a predefined cut-off value of 4. Moreover, the therapeutic benefit of lanreotide appeared to be independent of baseline NLR.

Similarly, Zhou et al. [40] reported that although lower NLR (the optimal cut-off value of NLR was 1.9) was associated with longer recurrence-free and overall survival in patients with resected PNENs, NLR did not remain an independent prognostic factor in multivariate analysis. Instead, the lymphocyte-to-monocyte ratio (LMR) was independently associated with recurrence-free survival.

These findings are important because they indicate that NLR should not be considered a universally applicable prognostic biomarker. Differences in patient selection, tumor grade, disease stage, treatment setting, sample size, duration of follow-up, and the cut-off values used to define high NLR may contribute to the heterogeneity of reported results [28,29,30,31,32,33,34,35,36,37,38,39,40].

### 5.8. Other Inflammatory Indices: PLR, LMR, MLR, SII, and Composite Prognostic Models

Recent studies further emphasize that NLR should be interpreted alongside other systemic inflammatory and nutritional indices. Lambrescu et al. [46], comparing 31 patients with NENs with 21 healthy controls, found significantly higher neutrophil counts, NLR, PLR, and systemic immune-inflammation index (SII) in patients with NENs. The pretreatment group also demonstrated significantly elevated neutrophil counts, NLR, and SII [46].

Yang et al. [48] found that LMR provided stronger independent prognostic information than NLR or PLR after surgery for PNETs. This observation is consistent with other studies showing that the relative prognostic performance of NLR, PLR, LMR, and related indices depends on the patient population and clinical endpoint [32,36,40,42,48].

More recently, Ye et al. [53] evaluated inflammation- and nutrition-related indices in 102 patients undergoing surgery for PNETs. The ROC-derived cut-offs were 49.675 for prognostic nutritional index (PNI), 3.413 for NLR, 2.467 for LMR, 256.155 for SII, and 150.319 for PLR. In multivariable analysis, PNI (HR 0.153, 95% CI 0.033–0.720; *p* = 0.017), NLR (HR 0.153, 95% CI 0.037–0.633; *p* = 0.010), and AJCC stage (HR 20.126, 95% CI 5.036–80.422; *p* < 0.001) were independent prognostic factors. A prognostic nomogram combining PNI, NLR, and AJCC stage was subsequently developed to predict 3-, 5-, and 10-year OS [53].

These findings suggest that NLR may have greater clinical utility when incorporated into multivariable or composite prognostic models rather than being used as an isolated biomarker.

### 5.9. Interpretation of the Current Evidence

The inclusion of recent studies [41,42,43,44,45,46,47,48,49,50,51,53] strengthens the overall evidence that elevated NLR is associated with advanced tumor stage, higher grade, recurrence, postoperative progression, and poorer survival in PNETs and broader GEP-NEN populations. In particular, the recent meta-analyses [45,50,51] provide more robust evidence that elevated NLR is associated with adverse OS and RFS outcomes. However, studies examining individual clinical settings continue to demonstrate substantial heterogeneity.

Importantly, NLR does not consistently retain independent prognostic significance after multivariable adjustment. For example, Yang et al. [48] found that LMR, rather than NLR, independently predicted postoperative progression, whereas Ocakli et al. [49] found no predictive value of NLR for lymph-node metastasis. Similarly, Ohlendorf et al. [44] demonstrated a relationship between baseline NLR and changes in tumor burden during PRRT but no independent association with PFS.

The available evidence therefore supports NLR as a complementary prognostic biomarker for disease outcomes in surgical series, and potentially as a predictive marker in advanced therapeutic settings, rather than a standalone replacement for established clinicopathological variables. Its interpretation must strictly distinguish between prognostic assessments (incorporating tumor grade, Ki-67, stage, and metastatic status) and predictive evaluations restricted to specific treatment settings [28,29,30,31,32,33,34,35,36,37,38,39,40,41,42,43,44,45,46,47,48,49,50,51,53].

The wide range of reported cut-off values—from approximately 2.28 and 2.62 in recent surgical cohorts [43,48] to ≥3.10 in meta-analytic stratification [51] and 3.413 in the prognostic nomogram study [53]—continues to limit the establishment of a universal threshold for clinical use.

#### 5.9.1. Potential Sources of Heterogeneity and Conflicting Findings

The discrepancies between studies reporting an independent prognostic association for NLR and those reporting null findings or stronger associations for other inflammatory indices should be interpreted in the context of methodological and clinical heterogeneity. Several factors may contribute to these differences. First, sample size and the number of outcome events may affect the precision of effect estimates and the stability of multivariable models. Studies with relatively few events may have limited power to detect an independent association, whereas models containing numerous covariates may be prone to overfitting. Conversely, statistically significant associations identified in small cohorts may be unstable and may overestimate the prognostic contribution of NLR.

Second, differences in patient selection, disease stage, tumor grade, primary tumor site, metastatic burden, and treatment setting may influence both baseline NLR and its relationship with outcomes. A marker associated with tumor burden or prognosis in one clinical context may have limited independent value in another, particularly when analyses combine patients undergoing surgery with those receiving systemic treatment or peptide receptor radionuclide therapy. The findings reported by Ohlendorf et al. [44], in which baseline NLR was associated with changes in tumor burden during PRRT but was not independently associated with progression-free survival, illustrate that an association with one disease-related measure does not necessarily translate into independent prognostic value for a time-to-event endpoint.

Third, residual confounding may arise from differences in the distribution or adjustment of established prognostic factors, including WHO grade, Ki-67 index, TNM stage, tumor size, and metastatic status. NLR may also reflect systemic inflammatory responses unrelated to tumor biology, and differences in the assessment or handling of such factors could contribute to inconsistent results. The observation that LMR, rather than NLR, retained independent prognostic significance in the study by Yang et al. [48] further suggests that the relative performance of inflammatory indices may depend on the cohort, the outcome examined, and the covariates included in the statistical model.

Finally, variation in endpoint definitions, follow-up duration, NLR measurement timing, cut-off selection, and statistical methods may limit comparability across studies. Data-driven cut-off selection and the use of different covariate-selection strategies can produce estimates that are sensitive to the particular dataset and modeling approach. Consequently, a nonsignificant result should not automatically be interpreted as evidence that NLR has no prognostic relevance, just as a statistically significant association in an individual cohort should not be regarded as proof of robust or clinically meaningful predictive value. Taken together, these discrepancies support a cautious interpretation of NLR as a potentially informative but context-dependent marker whose independent and incremental prognostic value remains insufficiently established.

#### 5.9.2. NLR Cut-Off Selection and Incremental Prognostic Value

An important limitation of the current evidence is the way in which NLR cut-off values have been selected. Across the reviewed studies, thresholds generally ranged from approximately 2 to 5, with several derived retrospectively using ROC analyses, median values, or outcome-oriented optimization [29,30,31,32,36,37,43,48,53]. Although such approaches may identify a threshold associated with outcomes in a particular cohort, data-driven cut-off selection can produce optimistic estimates of prognostic performance and increase the risk of overfitting, particularly in small datasets or when the number of outcome events is limited. Furthermore, thresholds optimized for different endpoints, including lymph-node involvement, recurrence, progression-free survival, and overall survival, are not necessarily interchangeable [29,30,31,32,37,48,53]. The apparent consistency of the association between elevated NLR and adverse outcomes should therefore be distinguished from validation of a reproducible clinical threshold. On the basis of the evidence reviewed here, a single universally applicable NLR cut-off cannot be recommended, and the extent to which proposed thresholds have been independently validated in external PNET cohorts remains unclear.

The evidence that NLR provides prognostic information beyond established clinicopathological factors is also incomplete. Some studies have reported independent associations between NLR and survival or recurrence after multivariable adjustment, and recent studies have explored models combining inflammatory indices with clinicopathological variables [29,31,37,48,53]. However, statistical independence in a regression model does not necessarily demonstrate a clinically meaningful improvement in individual risk prediction. The reviewed studies do not consistently establish whether adding NLR to models based on WHO grade, Ki-67 index, TNM stage, tumor size, and metastatic status improves discrimination, calibration, or clinical decision-making [38,48,53]. In particular, evidence of incremental performance would require comparison of otherwise identical models with and without NLR, external validation, and reporting of appropriate measures of discrimination and calibration [54]. Decision-curve analysis or other assessments of net clinical benefit would also help determine whether NLR-based risk classification could meaningfully inform surveillance or treatment decisions. Until such evidence is available, NLR is best regarded as a potentially complementary marker of systemic inflammation rather than a validated means of improving established prognostic models.

#### 5.9.3. Interpretation of Evidence According to Primary Site, Tumor Classification, and Treatment Setting

The available evidence on NLR in PNENs is heterogeneous and should be interpreted according to the population and clinical setting investigated. Studies restricted to PNETs provide the most direct evidence for the prognostic relevance of NLR in this population. These include cohorts of surgically treated patients and studies examining recurrence, pathological features, and survival [29,30,33,36,37,38,40,43,48]. Nevertheless, even among pancreatic-specific studies, differences in grade distribution, disease stage, eligibility criteria, and NLR thresholds may account for variation in the reported effect sizes.

A distinction between tumor grade and differentiation is particularly important. Well-differentiated PNETs, including grade 3 tumors, should not automatically be grouped with poorly differentiated pancreatic neuroendocrine carcinomas (PNECs). Studies that include both NETs and NECs, or that do not report differentiation separately, represent a biologically heterogeneous population and should be interpreted as evidence concerning pancreatic NENs more broadly rather than exclusively well-differentiated PNETs. Accordingly, studies with incompletely characterized histology cannot establish whether the observed association between NLR and outcome is consistent across these distinct entities.

A second source of heterogeneity is the inclusion of NENs arising from different primary sites. Studies of mixed GEP-NEN cohorts, including analyses derived from clinical trials and PRRT-treated populations, may support a general association between systemic inflammation and disease outcome. However, differences in tumor biology, natural history, grade distribution, and therapeutic sensitivity between pancreatic, intestinal, and other NENs limit the direct applicability of these findings to PanNETs [28,39,42,44,46,47,49]. When the number of patients with pancreatic primaries or a pancreatic-specific effect estimate is unavailable, the findings should be regarded as indirect evidence rather than confirmation of a prognostic association in PanNETs.

Finally, the prognostic significance of NLR may depend on the treatment setting. Surgical cohorts primarily address postoperative recurrence and survival, whereas studies of advanced disease treated with systemic agents or PRRT assess outcomes in different clinical contexts. Findings from resected PanNET cohorts therefore should not be assumed to apply equally to patients with unresectable or metastatic disease receiving systemic treatment. Similarly, results from mixed-primary PRRT cohorts cannot establish a treatment-specific prognostic effect in PanNETs unless pancreatic-specific analyses are reported. These distinctions are essential when comparing effect estimates and considering the potential clinical use of NLR.

### 5.10. Heterogeneity and Clinical Applicability

An important limitation of the available literature is the heterogeneity of the populations in which NLR has been investigated. Although several pancreatic-specific studies report associations between elevated NLR and adverse pathological features or survival [25,29,30,33,36,38,43,48], other studies include mixed pancreatic NEN histologies [31,37,40], multiple primary sites [28,42,46,49], or patients treated in substantially different clinical settings [32,39,42,44,47]. These populations should not be considered interchangeable. In particular, results from mixed GEP-NEN cohorts cannot be assumed to represent the prognostic performance of NLR in PanNETs, especially when the pancreatic subgroup size, histological differentiation, or subgroup-specific effect estimates are not reported [28,42,46,47,49,50].

Treatment setting further limits comparability. Evidence from patients undergoing curative-intent surgery addresses recurrence and postoperative survival [29,33,36,38,43,48], whereas studies of advanced disease receiving systemic therapy or peptide receptor radionuclide therapy (PRRT) assess outcomes in different clinical circumstances [39,42,44,47]. The association between NLR and outcome may consequently differ according to disease extent, treatment selection, and the endpoint evaluated. Meta-analyses may increase statistical precision, but they do not eliminate these limitations when their constituent studies differ in primary site, histological classification, grade, stage, treatment, and NLR cut-off [35,45,50,51].

The true prognostic independence of NLR remains highly variable when studies are compared with respect to follow-up duration and the specific covariates included in multivariable analyses [28,29,30,31,32,33]. While some cohorts feature prolonged observation periods—such as the surgical series by Tong et al. [31], which presents a mean RFS tracking exceeding 5 years (mean RFS of 61.1 ± 4.4 months in the high-NLR group vs. 63.8 ± 2.9 months in the low-NLR group, respectively) during the median follow-up of 31 (range 3–84) months—others rely on significantly shorter evaluation windows. Notably, the prospective study by Gaitanidis et al. [32] carries a median follow-up of only 15 (range 12–73) months, and the cohort by Salman et al. [28] focuses on a median PFS prediction of just 11.5 months in patients with a median NLR of 2.17. Such limited timelines may be insufficient to fully capture the true, long-term natural history of well-differentiated PNETs. More importantly, there is no consensus on the adjusters used in multivariable regressions [28,29,30,31,32,33]. In several cohorts, NLR loses its independent predictive value for survival or recurrence once robust clinical variables—such as WHO histological grade, Ki-67 index, TNM stage, lymphovascular invasion, or alternative indices like the LMR—are introduced into the statistical model. This inconsistency indicates that the reported effect sizes are highly dependent on model architecture and covariate selection rather than reflecting a universal biological truth.

Accordingly, the most directly applicable evidence currently comes from studies restricted to pancreatic primaries, particularly cohorts with clearly documented tumor differentiation and treatment setting [25,29,30,33,36,43,48]. Results from broader NEN populations should be regarded as supportive, hypothesis-generating evidence unless confirmed in pancreatic-specific analyses [28,42,44,46,47,49,50]. 

However, even within these targeted cohorts, the clinical specificity of NLR remains obscured by inconsistent accounting for non-oncological systemic stress. Because neutrophils and lymphocytes respond dynamically to subclinical infections, corticosteroid therapy, or acute physiological stress, an elevated preoperative NLR cannot be assumed to reflect tumor-associated inflammation exclusively. Most reviewed retrospective studies failed to apply strict clinical or pharmacological exclusion criteria during data collection, meaning that a high baseline NLR may often be a secondary artifact rather than a true representation of the tumor microenvironment [48,52]. 

Furthermore, variation in NLR thresholds and the inconsistent demonstration of independent prognostic value indicate that NLR should not currently be used as a stand-alone prognostic tool [36,40,48]. Its potential role is as an adjunct to established factors, including differentiation, WHO grade, Ki-67 index, TNM stage, lymph-node involvement, and distant metastases [29,30,33,38,43,48]. In this context, ensuring the acquisition of good-quality tissue samples continues to represent a vital benchmark for uncovering potential tissue-based prognostic biomarkers, particularly since accurate preoperative sampling is essential to minimize grading discrepancies [27].

### 5.11. Summary of the Current Evidence

Overall, the current evidence [28,29,30,31,32,33,34,35,36,37,38,39,40,41,42,43,44,45,46,47,48,49,50,51,53] indicates a broad and increasingly well-supported association between systemic inflammatory markers and PNET aggressiveness and prognosis. Elevated NLR is generally associated with higher tumor grade, more advanced stage, recurrence or postoperative progression, and worse survival, with the strongest overall support provided by recent meta-analyses [45,50,51]. Nevertheless, its performance is not uniform across all populations or treatment settings.

Other indices, particularly LMR, PLR, SII, and nutrition-related markers such as PNI, may provide additional or occasionally superior prognostic information [32,40,42,46,47,48,53]. Accordingly, the current direction of research appears to favor integrated prognostic models combining systemic inflammation with established pathological and staging variables, rather than reliance on NLR alone [48,53].

The summary of studies evaluating NLR in PNENs and GEP-NENs is presented in Table 2. 

## 6. Clinical Utility

The available evidence suggests that NLR may have potential clinical utility as a simple, inexpensive, and readily available biomarker for risk assessment in patients with PNETs. Because NLR is calculated from routinely obtained peripheral blood counts, it could potentially be incorporated into the preoperative evaluation without requiring additional invasive procedures or specialized laboratory testing. The observed associations between elevated NLR and higher tumor grade, advanced stage, metastatic disease, recurrence, and unfavorable survival outcomes suggest that NLR may contribute to preoperative risk stratification [28,29,30,31,32,36,37,41,43,45,48,50,51].

In particular, preoperative NLR may help identify patients with a potentially more aggressive tumor phenotype. Several studies have demonstrated associations between elevated NLR and higher WHO grade, increased Ki-67 index, larger tumor size, advanced TNM stage, and lymph-node or distant metastases [29,30,31,37,41,45,48]. Therefore, when interpreted together with radiological findings and established pathological or clinical variables, a high NLR could provide additional information regarding the probability of biologically aggressive disease before surgery.

NLR may also assist in identifying patients at increased risk of recurrence following curative-intent surgery. Elevated preoperative NLR has been associated with shorter RFS or DFS and with a higher risk of postoperative recurrence in several cohorts [29,31,36,37,43,45,50,51]. For example, Arima et al. [29] identified NLR ≥ 2.4 as an independent predictor of postoperative recurrence, whereas Miura et al. [43] reported that an NLR > 2.62 independently predicted recurrence after curative surgery. Recent meta-analyses have further supported the association between high NLR and poorer RFS [45,50,51]. Consequently, NLR could potentially contribute to the identification of patients who may benefit from closer clinical follow-up after surgery.

Another potential application is the planning of postoperative surveillance. Patients with elevated preoperative NLR, particularly when additional high-risk features are present, may represent a subgroup with a greater likelihood of recurrence or disease progression. NLR could therefore be incorporated into a broader risk assessment model to support decisions regarding the intensity and frequency of postoperative imaging and clinical monitoring. However, no reviewed study has established a specific NLR-guided surveillance protocol. Thus, this potential application should currently be regarded as a hypothesis-generating use rather than a validated clinical strategy [28,29,30,31,32,33,34,35,36,37,38,39,40,41,42,43,44,45,46,47,48,49,50,51,53]. NLR could therefore be incorporated into a broader risk assessment model to support decisions regarding the intensity and frequency of postoperative imaging and clinical monitoring. However, no reviewed study has established a specific NLR-guided surveillance protocol. Thus, this potential application should currently be regarded as a hypothesis-generating use rather than a validated clinical strategy [28,29,30,31,32,33,34,35,36,37,38,39,40,41,42,43,44,45,46,47,48,49,50,51,53].

The most appropriate role of NLR may be as a complementary biomarker rather than an independent substitute for established prognostic factors. Ki-67 proliferation index, WHO grade, TNM stage, lymph-node involvement, and distant metastasis remain the principal determinants of risk assessment in PNETs. The available evidence suggests that NLR may provide additional information regarding the systemic inflammatory response and host–tumor interaction, which is not fully captured by conventional pathological staging alone [28,29,30,31,32,33,34,35,36,37,38,39,40,41,42,43,44,45,46,47,48,49,50,51,53]. Recent studies also suggest that combining NLR with other inflammatory, nutritional, and clinicopathological variables may improve prognostic performance [38,48,53].

Nevertheless, the clinical implementation of NLR remains limited by the considerable variability in reported cut-off values. Across the reviewed studies, thresholds ranged approximately from 2 to 5, although individual analyses identified values such as 2.28, 2.3, 2.4, 2.62, 3.41, and 3.7, depending on the population and clinical endpoint [29,30,31,32,36,37,43,48]. This heterogeneity currently prevents the recommendation of a universal threshold for routine clinical decision-making. In addition, NLR may be influenced by inflammatory conditions unrelated to the tumor, which further limits its specificity. Therefore, NLR should be interpreted in the context of the patient’s overall clinical condition and should not be used as an isolated determinant of treatment or surveillance decisions.

Clinical utility of NLR is summarized in Table 3.

## 7. Limitations

The interpretation of the current literature is subject to several important limitations. First, most available evidence is derived from retrospective, single-center studies [28,29,30,31,32,33,34,35,36,37,38,39,40,41,43,44,46,47,48,49,53]. Retrospective designs are inherently susceptible to selection bias, incomplete data collection, and differences in patient management. Although several meta-analyses have strengthened the overall evidence [45,50,51], the quality of their conclusions remains dependent on the methodological characteristics and heterogeneity of the included studies.

A further limitation is the substantial heterogeneity of the study populations. While some investigations focused specifically on surgically treated PNETs or PNENs, others included patients with broader GEP-NENs or mixed populations with different primary tumor sites, grades, disease stages, and treatment strategies [32,42,44,46,47,49,50]. Consequently, findings obtained in mixed GEP-NEN cohorts cannot always be directly extrapolated to patients with PNETs. Similarly, the prognostic role of NLR may differ between patients undergoing curative surgery, those with metastatic disease, and those receiving systemic therapy or peptide receptor radionuclide therapy.

The lack of a standardized NLR threshold represents another major limitation. Different studies used different approaches to determine the optimal cut-off value, including ROC analysis, median values, predefined thresholds, or values derived from survival analyses. As a result, the reported thresholds vary considerably, ranging approximately from 2 to 5 [29,30,31,32,36,37,43,48,50,51,53]. This variability makes direct comparison between studies difficult and currently limits the clinical applicability of a single universal NLR cut-off.

Many thresholds were selected retrospectively within the same cohorts in which their prognostic associations were evaluated [29,30,31,32,36,37,43,48,53]. This approach may lead to optimistic estimates of effect and predictive performance, particularly in small retrospective studies, and limits the reproducibility of the proposed cut-offs. The available evidence does not establish that the thresholds identified in individual cohorts are consistently transferable to independent populations with different disease stages, histological characteristics, treatment settings, and outcome definitions. In addition, the independent prognostic significance of NLR should not be equated with incremental predictive value. Although some studies report statistically significant associations after multivariable adjustment, the available literature provides limited and inconsistent evidence that adding NLR to established models improves discrimination, calibration, or clinical net benefit [38,48,53]. Formal comparisons of models with and without NLR, followed by external validation, are needed before its added value for patient-level risk prediction can be established [54].

NLR is also susceptible to confounding by factors unrelated to tumor biology. Infection, acute or chronic inflammatory conditions, and comorbidities may alter neutrophil and lymphocyte counts and consequently affect the calculated NLR. Therefore, an elevated NLR cannot be assumed to reflect tumor-associated systemic inflammation exclusively. This limitation is particularly relevant in retrospective studies, in which information regarding concurrent inflammatory conditions or other potential confounders may not have been collected systematically.

A major methodological challenge in the current literature is the lack of standardized reporting regarding the exact timing of peripheral blood sampling and the methods used for cutoff selection [28,29,30,31,32,33,34,35,36,37,38,39,40,41,43,48,49,53]. While most surgical series analyze “preoperative” NLR, the exact window ranges from the day of admission to several weeks prior to resection. Furthermore, the thresholds defining a “high” NLR are predominantly data-driven—established retrospectively via ROC curve analysis or median values within individual, limited sample sizes—rather than validated prospectively in independent cohorts [28,29,30,31,32,33,34,35,36,37,38,39,40,41,43,48,53]. This statistical dependency artificially inflates prognostic performance and hinders cross-study reproducibility.

Crucially, the clinical specificity of NLR remains obscured by the inconsistent tracking of potential systemic confounders. Because neutrophils and lymphocytes dynamically respond to non-oncological stress, an elevated NLR can be heavily biased by subclinical infections, corticosteroid therapy, or active concomitant inflammatory and autoimmune conditions. Despite this biological reality, the vast majority of reviewed retrospective cohorts failed to systematically describe or apply strict exclusion criteria for these covariates during data collection [28,29,30,31,32,33,34,35,36,37,38,39,40,41,43,44,46,47,48,49,53]. Consequently, it remains uncertain whether a high baseline NLR truly reflects a tumor-promoting immunosuppressive microenvironment or is merely an artifact of secondary, unrelated inflammatory responses [48,52].

Furthermore, a critical appraisal of the available meta-analytic evidence reveals a substantial, yet rarely acknowledged, overlap among primary patient cohorts. For instance, large multicenter databases, such as the U.S. Neuroendocrine Tumor Study Group (US-NETSG) or single-center series from major tertiary institutions, are repeatedly extracted and pooled across different meta-analyses published between 2018 and 2025 [35,45,50,51]. This redundancy means that a significant portion of the primary patient data is counted multiple times across the literature, specifically within the pooled analyses by Oh et al. [45], Worapongpaiboon et al. [51], and Wang et al. [50]. Readers must be cautioned that such repeated inclusion does not constitute independent validation; instead, it artificially narrows confidence intervals and creates an illusion of robust consensus that masks the underlying heterogeneity of the original source studies.

Prospective validation remains limited. Although prospective evidence is available in selected settings, including studies of advanced disease and systemic treatment [32,42], most data concerning the prognostic value of NLR in surgically treated PNETs remain retrospective. Furthermore, few studies have evaluated whether the incorporation of NLR into clinical decision-making actually improves patient outcomes. Future prospective, multicenter studies should therefore evaluate standardized NLR measurement, predefined cut-off values, and its incremental value beyond established prognostic factors such as Ki-67, WHO grade, and TNM stage.

A further important limitation of the current evidence is the scarcity of data regarding the relationship between preoperative NLR and postoperative complications. Most studies evaluating systemic inflammatory markers in PNETs have focused primarily on tumor grade, stage, recurrence, progression, and long-term survival rather than short-term surgical outcomes [28,29,30,31,32,33,34,35,36,37,38,39,40,41,42,43,44,45,46,47,48,49,50,51,53]. Although some studies reported associations between elevated NLR and adverse pathological features or greater intraoperative blood loss, the ability of NLR to predict specific postoperative complications has not been systematically established.

A critical and largely unacknowledged methodological limitation within the available literature is the substantial overlap of primary patient cohorts across multiple published meta-analyses. Large multicenter databases, such as those from the U.S. Neuroendocrine Tumor Study Group (US-NETSG) [36], or specific major tertiary centers, have been repeatedly extracted and pooled in systematic reviews published between 2018 and 2025. This redundancy means that a significant volume of identical patient data is counted multiple times across different papers—specifically within the pooled data presented by Oh et al. [45], Worapongpaiboon et al. [51], and Wang et al. [50].

This represents a relevant knowledge gap because postoperative morbidity remains substantial after pancreatic surgery for PNETs. Clinically relevant postoperative pancreatic fistula (CR-POPF) is one of the most common complications, with its incidence varying according to the type of surgical procedure; a systematic review reported pancreatic fistula rates ranging from approximately 14% after distal pancreatectomy and pancreatoduodenectomy to 45% after enucleation and 58% after central pancreatectomy. Moreover, POPF is the main source of postoperative morbidity, and is associated with numerous further complications. However, these data describe the incidence of complications and do not establish whether NLR or other systemic inflammatory markers can predict their occurrence [54,55,56,57,58].

Importantly, inflammatory markers may themselves be influenced by perioperative factors. Infection, systemic inflammation, surgical stress, medications, and other comorbidities can affect neutrophil and lymphocyte counts and may therefore confound the relationship between NLR and postoperative outcomes. Consequently, the current evidence is insufficient to determine whether an elevated preoperative NLR represents an independent predictor of postoperative morbidity or merely reflects other patient- or tumor-related factors [48,52].

Given these clear gaps, any clinical application of preoperative NLR in forecasting short-term surgical morbidity or tailoring perioperative strategies must be explicitly treated as an unvalidated research hypothesis rather than an established protocol [48,54]. While statistical associations with long-term survival are widely documented [28,30,33,36,43,45,50,51,53], the available data consistently fail to demonstrate that NLR provides any incremental predictive value or independent clinical utility regarding acute postoperative complications [48,52]. Future prospective multicenter trials with standardized timing of blood sampling and rigorous exclusion of non-oncological confounders are strictly required to establish whether NLR can add reproducible predictive power to conventional surgical risk models [38,48,53], and whether its clinical implementation can translate into objective improvements in patient outcomes [54,55,56,57,58].

Overall, these limitations indicate that NLR should currently be regarded as a promising adjunctive biomarker rather than a fully validated tool for routine clinical decision-making. Further prospective validation and the development of standardized, integrated prognostic models are required before NLR-guided risk stratification or surveillance strategies can be formally implemented in clinical practice. 

## 8. Future Perspectives and Directions

Future research should focus primarily on establishing the clinical role of NLR within standardized prognostic models for patients with PNETs. Although the available evidence consistently suggests an association between elevated NLR and adverse clinicopathological features and survival outcomes, substantial heterogeneity remains regarding patient populations, treatment settings, endpoints, and NLR cut-off values [28,29,30,31,32,33,34,35,36,37,38,39,40,41,42,43,44,45,46,47,48,49,50,51,53]. Large prospective, multicenter studies focusing specifically on PNETs are therefore needed to validate the prognostic value of NLR and determine whether a clinically applicable threshold can be established.

An important direction for future research is the standardization of NLR assessment. Future studies should use predefined blood sampling time points, clearly defined exclusion criteria for acute infection or inflammatory conditions, and standardized approaches to the management of potential confounders. The determination of NLR thresholds should preferably be based on prospective validation rather than data-driven cut-off selection within individual cohorts. Such standardization would improve comparability between studies and facilitate the development of clinically useful risk categories.

Future investigations should also determine the incremental prognostic value of NLR beyond established clinicopathological factors. Rather than evaluating NLR as an isolated biomarker, studies should assess whether its addition improves the predictive performance of models based on WHO grade, Ki-67 index, TNM stage, tumor size, lymph-node status, and distant metastases. Recent studies combining inflammatory markers with clinicopathological or nutritional parameters suggest that integrated models may provide better prognostic stratification than individual variables alone [38,48,53]. However, these models require external validation before they can be considered for routine clinical use [54].

Future validation studies should prespecify the NLR threshold or evaluate NLR as a continuous variable to reduce information loss and avoid dependence on cohort-specific dichotomization. Where a cut-off is developed, its performance should be assessed in an independent cohort rather than solely in the derivation dataset. Studies should compare a baseline model incorporating established prognostic factors, including WHO grade, Ki-67 index, and TNM stage, with an otherwise identical model that additionally includes NLR. This approach would extend previous work exploring integrated models incorporating inflammatory and clinicopathological variables [38,48,53]. Relevant outcomes should include changes in discrimination, calibration across clinically relevant time points, and clinical net benefit at plausible decision thresholds. Internal validation using appropriate resampling methods may help quantify optimism during model development, but should not be considered a substitute for external validation [54]. These steps would clarify whether NLR provides reproducible prognostic information and whether that information is sufficiently useful to influence surveillance or treatment decisions in clinical practice.

Another promising direction is the development of multivariable and composite biomarkers incorporating NLR together with PLR, LMR, MLR, SII, PNI, or other markers of systemic inflammation and nutritional status. The available literature indicates that the relative prognostic performance of individual inflammatory indices varies between patient populations and clinical settings [32,36,38,40,42,46,47,48,53]. Therefore, combining several complementary parameters may better capture the complex interaction between tumor biology, systemic inflammation, immune status, and host nutritional condition.

The role of NLR in treatment-specific prognostication also requires further investigation. Available studies suggest that the significance of NLR may differ between patients undergoing curative surgery, those receiving systemic therapies, and those treated with PRRT [32,42,44,47]. Future prospective studies should therefore evaluate whether baseline or dynamic changes in NLR can predict treatment response, disease progression, or survival in specific therapeutic settings. Repeated measurements may be particularly relevant, as longitudinal changes in inflammatory status could potentially provide information beyond a single baseline assessment.

Another important research area is the potential application of NLR to individualized postoperative surveillance. Future studies should investigate whether combining preoperative or postoperative NLR with established recurrence risk factors can identify subgroups requiring different surveillance intensity. Such approaches should not only demonstrate an association with recurrence but also show that biomarker-guided follow-up strategies improve clinically relevant outcomes and can be implemented efficiently.

Additionally, future studies should clarify the biological mechanisms underlying the association between elevated NLR and aggressive PNET behavior. Integrating peripheral blood inflammatory markers with tumor immune microenvironment characteristics, pathological findings, and molecular or genomic data may help explain why NLR has prognostic significance in some populations but not in others. This approach could also identify patient subgroups in which systemic inflammatory markers are particularly informative.

Finally, an important and insufficiently explored direction for future research is the potential association between preoperative systemic inflammatory markers and postoperative complications. While elevated NLR has been extensively investigated as a predictor of tumor aggressiveness, recurrence, progression, and survival, its potential value for predicting short-term surgical morbidity in patients undergoing resection for PNETs remains largely unknown [28,29,30,31,32,33,34,35,36,37,38,39,40,41,42,43,44,45,46,47,48,49,50,51,53]. This represents a significant gap because postoperative complications, particularly CR-POPF, postoperative acute pancreatitis (POAP), delayed gastric emptying (DGE), postoperative hemorrhage, and infectious complications, may substantially influence recovery, length of hospitalization, subsequent treatment, and overall patient outcomes [54]. 

Future prospective studies should therefore specifically investigate whether preoperative NLR, alone or in combination with PLR, LMR, SII, nutritional indices, and established surgical risk factors, can predict postoperative complications. Such analyses should evaluate standardized outcomes, including overall postoperative morbidity, severe complications according to the Clavien–Dindo classification, clinically relevant postoperative pancreatic fistula, delayed gastric emptying, postoperative hemorrhage, infectious complications, reoperation, length of hospital stay, and postoperative mortality.

Importantly, future studies must rigorously distinguish between the prognostic role of NLR for long-term survival outcomes, its potential predictive value regarding systemic or targeted treatment response, and its capability to forecast short-term postoperative complications. These represent fundamentally distinct clinical questions requiring independent validation models and tailored threshold definitions. Because NLR can be influenced by pre-existing infection, chronic inflammatory conditions, medication use, and other comorbidities, future studies should carefully control for these confounding factors and use standardized preoperative blood sampling protocols. 

Ultimately, if an independent association between NLR and postoperative morbidity is confirmed, NLR could potentially become part of a broader preoperative surgical risk assessment model. Such a nomogram could combine systemic inflammatory markers with established clinical and operative risk factors to improve patient selection, perioperative optimization, counseling, and postoperative monitoring. At present, however, the evidence is too limited to recommend NLR as a predictor of postoperative complications in routine clinical practice, and this question should be considered an important priority for future prospective PNET-specific research.

The absolute next frontier for NLR research must shift away from iterative, single-center retrospective survival analyses. To move from a laboratory curiosity to a clinically actionable index, future prospective trials must explicitly evaluate whether the clinical implementation of NLR-guided surveillance schedules, personalized follow-up intensities, or treatment selection algorithms actually results in a demonstrable, objective improvement in individual patient outcomes [32,38,42,47,53].

Overall, future research should move beyond repeated retrospective evaluations of NLR as a single prognostic variable. The next step should be prospective validation of standardized, PNET-specific, and clinically applicable prognostic models integrating systemic inflammatory markers with established pathological and clinical factors, as well as searching for potential associations between preoperative systemic inflammatory markers and postoperative complications to construct a predictive score for postoperative complications including NLR and other clinicopathological and laboratory parameters. Such studies may determine whether NLR can progress from an easily measurable research biomarker to a useful component of individualized risk stratification, postoperative surveillance, and treatment planning.

Future studies should also be designed to address the methodological sources of heterogeneity that may explain the conflicting results reported to date. Prospective multicenter cohorts with clearly defined eligibility criteria, standardized timing of NLR measurement, prespecified outcomes, and adequate follow-up would improve comparability across studies. Sample-size calculations should account for the expected number of outcome events and the complexity of the planned statistical models. Analyses should prespecify clinically relevant confounders, including WHO grade, Ki-67 index, TNM stage, tumor burden, and treatment setting, while limiting model complexity to reduce overfitting. The handling of missing data, variable selection, and cut-off determination should be transparently reported, and data-derived thresholds should be evaluated in independent cohorts. Where feasible, studies should also investigate whether the prognostic performance of NLR differs across clinically relevant subgroups or treatment settings. A more consistent design and reporting framework would help distinguish genuine differences in the prognostic relevance of NLR from variation attributable to study methods and population characteristics.

## 9. Conclusions

The available evidence suggests that the NLR is a promising and readily available biomarker associated with the biological aggressiveness and prognosis of PNETs. Across the reviewed studies, elevated NLR was generally associated with higher tumor grade, increased Ki-67 proliferative activity, larger tumor size, advanced TNM stage, lymph-node or distant metastasis, and an increased risk of recurrence [28,29,30,31,32,33,34,35,36,37,38]. Most studies also demonstrated an association between elevated NLR and poorer survival outcomes, including overall survival, recurrence-free survival, disease-free survival, and progression-free survival [28,29,30,31,32,33,34,35,36,37,38].

However, the prognostic value of NLR was not confirmed uniformly across all patient populations and clinical settings. In some studies, NLR did not remain an independent prognostic factor after adjustment for established clinicopathological variables, while other inflammatory indices, particularly LMR, demonstrated independent prognostic significance [39,40]. Moreover, substantial heterogeneity exists with respect to study design, patient selection, tumor stage and grade, treatment setting, follow-up duration, and the NLR cut-off values applied. Consequently, a universally applicable threshold for defining elevated NLR has not yet been established.

Other systemic inflammatory indices, including PLR, LMR, MLR, MPV, and composite markers such as PD-NLR, may provide additional prognostic information [28,32,36,38,40]. Their relative clinical value, however, remains uncertain, and current evidence does not support the superiority of a single inflammatory marker across all clinical settings.

Overall, NLR should currently be considered a potentially useful complementary biomarker rather than a replacement for established prognostic factors, including WHO grade, Ki-67 proliferation index, TNM stage, lymph-node involvement, and distant metastasis. Its greatest potential may lie in improving preoperative risk stratification because it can be easily calculated from routine blood tests. Future prospective, multicenter studies using standardized definitions and clinically validated cut-off values are needed to determine whether NLR, alone or in combination with other inflammatory and clinicopathological parameters, can be incorporated into routine prognostic models for patients with PNETs [28,29,30,31,32,33,34,35,36,37,38,39,40]. 

Consequently, any proposed clinical applications of NLR in defining treatment selection, determining surveillance intensity, or predicting postoperative outcomes must be explicitly categorized as investigational. At present, NLR represents a promising candidate biomarker whose independent clinical validity awaits prospective confirmation in standardized, pancreatic-specific trials [28,29,30,31,32,33,34,35,36,37,38,39,40,48].

Overall, the available evidence suggests that elevated NLR may be associated with adverse outcomes in some patients with PNENs; however, the consistency and independence of this association vary across studies and clinical settings. Negative findings, including studies in which other inflammatory indices showed stronger independent prognostic associations, or NLR was not independently associated with progression-free survival, underscore the need to avoid overgeneralization. The current literature does not yet establish a reproducible NLR threshold or consistently demonstrate incremental prognostic value beyond established clinicopathological factors. NLR should therefore be considered a candidate complementary biomarker requiring further validation, rather than a stand-alone or clinically validated prognostic tool.

## Figures and Tables

**Table 1:** **The WHO classification and grading of pancreatic neuroendocrine neoplasms (PNENs)** [1,2].

Terminology	Differentiation	Grading	Mitotic Count	KI-67 Index
NET G1 NET G2 NET G3 NEC small-cell type (SCNEC) NEC large-cell type (LCNEC) MiNEN	Well differentiated Poorly differentiated Well or poorly differentiated	Low Intermediate High High High Various	<2 2–20 >20 >20 >20 Various	<3% 3–20% >20% >20% >20% Various

**Abbreviations:** WHO, World Health Organization; Neuroendocrine tumor; NEC, Neuroendocrine carcinoma; SCNEC, Small-cell neuroendocrine carcinoma; LCNEC, Large-cell neuroendocrine carcinoma; MiNEN, Mixed neuroendocrine–non-neuroendocrine neoplasm. Mitotic counts are to be expressed as the number of mitoses/2 mm^2^ as determined by counting in 50 fields of 0.2 mm^2^ (i.e., in a total area of 10 mm^2^); the Ki-67 proliferation index value is determined by counting at least 500 cells in the regions of highest labelling (hot-spots), which are identified at scanning magnification; the final grade is based on whichever of the two proliferation indexes places the neoplasm in the higher-grade category [1].

**Table 2:** **Summary of studies evaluating NLR and other systemic inflammatory markers in PNENs and GEP-NENs.**

First Author [Ref.]	Publication Year	Country	Study Design	Population	Differentiation/Grading	AJCC TNM Staging/Extend of Disease	Patients Overall, n Patients with Primary Pancreatic Site, n	Treatment Setting	NLR Cut-Off	Effect Size	Key Findings	Applicability to PNETs
Salman [28]	2016	Turkey	Single-center retrospective	GEP-NETs PNET 50 (37.9%) GE-NET 82 (62.1%)	G1 44 G2 41 G3 47	I 13 II 26 III 35 IV 58	132 50	NR	2.17 (median)	r = −0.862 for NLR vs. PFS; AUC = 0.94	Higher NLR was associated with higher tumor grade, advanced TNM stage, metastatic disease, and shorter PFS.	Indirect; mixed GEP-NET cohort
Arima [29]	2017	Japan	Single-center retrospective	PNETs	G1 46 G2 9 G3 3	Postoperative recurrence and liver metastases assessed	58 58	Preoperative assessment and curative resection	2.4	Recurrence: HR 6.012; postoperative liver metastases: HR 7.570	NLR ≥ 2.4 independently predicted postoperative recurrence and liver metastases and was associated with shorter OS and RFS.	Direct; pancreatic surgical cohort
Luo [30]	2017	China	Single-center retrospective	PNETs	G1 63 G2 58 G3 15	I-II 107 III-IV 58	165 165	NR	2.4	OS, multivariable HR 3.60	NLR > 2.4 was associated with larger tumors, advanced TNM stage, higher grade, and independently poorer OS.	Direct; pathologically confirmed pancreatic cohort
Zhou [33]	2017	China	Single-center retrospective with healthy controls	Resected PNETs	G1 73 G2 76 G3 23	I-II 150 III-IV 22	172 PNETs + 172 controls	Preoperative assessment and curative resection	2.31	OS: HR 4.471; DFS: HR 2.531	Elevated preoperative NLR independently predicted poorer OS and DFS and improved conventional risk stratification.	Direct; pancreatic surgical cohort
Zhou [34]	2017	China	Single-center retrospective	Non-functional PNETs after curative resection	G1 24 G2 56 G3 21	N1 27 M1 15	101 101	Preoperative assessment and curative resection	1.80	LN metastases: HR 6.218; AUC = 0.734	NLR ≥ 1.80 independently predicted lymph-node metastases and was associated with shorter DFS.	Direct; pancreatic surgical cohort
Tong [31]	2018	China	Single-center retrospective	Resectable PNENs	G1 52 G2 32 NEC G3 11	T1 34 T2 28 T3 21 T4 12 N0 80 N1 15 M0 80 M1 15	95 95	Preoperative assessment and curative resection	2.056 for LN metastases; 1.40 for RFS	LN metastases: HR 6.740	High NLR was associated with advanced T stage, higher grade, tumor thrombus, LN metastases, and shorter RFS.	Direct; pancreatic surgical cohort, but includes NEC G3; not exclusively well-differentiated PNETs
Gaitanidis [32]	2018	International, multicenter	Prospective	PNETs; surgical (*n* = 34) and non-surgical (*n* = 63) subgroups	G1 41 G2 18 G3 2	Locoregional 39 Distant metastases 24 N0 22 N1 12 M0 30 M1 4	97 97	Curative resection and non-surgical management	2.3 (surgical cohort); 2.5 (non-surgical cohort)	Surgical PFS: HR 2.53	NLR > 2.3 independently predicted worse PFS after curative resection; in non-surgical patients NLR was not independently prognostic.	Direct; results depend on treatment setting
Zhou [35]	2018	China	Meta-analysis	GEP-NETs; predominantly PNET studies	Mixed disease grade	Mixed disease stages	724 from 8 studies	Pretreatment assessment Mixed treatment settings	Study-specific	PNET OS: pooled HR 4.21; PNET RFS: pooled HR 5.37	Elevated NLR was associated with significantly poorer OS and RFS, including in the PNET subgroup.	Direct and indirect evidence; predominantly pancreatic studies
Panni [36]	2019	USA	Single-center retrospective	Resectable PNETs	G1 305 G2 152 G3 20	Tumor size 3.381 ± 2.9 cm N0 497 N1 123 M0 537 M1 83	620 620	Preoperative assessment and curative resection	3.7	RFS: HR 1.79; OS: HR 2.04	NLR ≥3.7 independently predicted worse RFS and OS after adjustment for clinicopathological factors.	Direct; large pancreatic surgical cohort
Harimoto [37]	2019	Japan	Single-center retrospective	PNETs undergoing potentially curative pancreatic resection	G1 34 G2 17 G3 4	Tumor size 2.5 ± 1.45 cm N0 45 N1 10	55 55	Curative resection	3.41	AUC = 0.795 for recurrence	High NLR was associated with higher Ki-67, mitotic count, grade, LN metastases, lymphatic/neural invasion, blood loss, and poorer RFS.	Direct; poorly differentiated NEC excluded
Zhou [38]	2019	China	Single-center retrospective	PNETs of the pancreatic head after curative resection	G1 28 G2 26 G3 10	T 1-2 49 T 3-4 15 N0 49 N1 15 M0 61 M1 3	64 64	Preoperative assessment and curative resection	3.13; incorporated into PD-NLR score	NLR and OS: HR 8.837; PD-NLR and OS: HR 16.159; DFS: HR 7.356	High NLR and PD-NLR were associated with aggressive pathological features and independently poorer OS and DFS.	Direct; pancreatic surgical cohort
Grenader [39]	2020	International, multicenter	Post-hoc analysis of randomized double-blind CLARINET trial	Advanced intestinal and pancreatic NETs, G1–G2	G1 138 G2 61 Missing 2	Advanced disease (metastatic or locally advanced); detailed stage distribution NR	201 88	Lanreotide versus placebo	4; also tertiles	No significant prognostic HR reported	NLR was not associated with PFS, and the benefit of lanreotide was independent of baseline NLR.	Indirect; mixed-primary evidence; the pancreatic-specific effect cannot be determined
Zhou [40]	2020	China	Single-center retrospective	Resected pNENs	G1 73 G2 88 G3 13	I 54 II 96 III 15 IV 9	174 174	Preoperative assessment and curative resection	1.9	NLR not independent; LMR HR 0.30 for RFS	Lower NLR was associated with longer RFS and OS, but only LMR remained an independent predictor of RFS.	Direct; pancreatic surgical cohort
Dikmen [41]	2020	Turkey	Single-center retrospective case–control	PNETs and healthy controls	G1 26 G2 3 G3 15	I 25 II 9 III 3 IV 9	44 PNETs + 44 controls	Preoperative assessment and curative resection	No prognostic cut-off; median NLR 2.4	*p* = 0.001 for LN metastases	NLR increased with ENETS and TNM stage and was significantly higher in patients with LN metastases.	Direct PNET evidence for disease extent; not a survival prognostic study
Chan [42]	2021	International, multicenter	Pooled analysis of two phase III randomized trials	GI, pancreatic, and lung NETs; everolimus/placebo	Grade and differentiation distribution NR	Advanced disease; N0 234 N1 118	712 410	Everolimus versus placebo	2.58	PFS: HR 0.75 for lower vs. higher NLR	Higher NLR was associated with shorter median PFS in the pooled population and treatment subgroups.	Indirect; mixed-primary evidence;
Miura [43]	2021	Japan	Single-center retrospective	Well-differentiated PNETs undergoing curative surgery	G1 73 G2 45 G3 2	I 74 II 29 III 19	120 120	Preoperative assessment and curative resection	2.62	Recurrence: HR 3.49; non-functional tumors RFS: HR 4.95	High NLR independently predicted recurrence, particularly in non-functional PNETs.	Direct; well-differentiated pancreatic surgical cohort
Ohlendorf [44]	2021	Germany	Single-center retrospective observational cohort	Advanced GEP-NETs treated with PRRT	G1 7 G2 21 G3 1 Unknown 4	Advanced disease	33 10	PRRT	Not specified	Tumor burden change: *p* = 0.004; PFS HR 0.86	Baseline NLR was associated with changes in tumor burden during PRRT but not with PFS.	Indirect; mixed-primary treatment-response evidence; pancreatic-specific applicability is uncertain
Oh [45]	2022	Meta-analysis	Meta-analysis 9 studies	PNETs	G1 648 G2 400 G3 63	Heterogeneous across included studies	1121 NR	Mixed treatment settings across included studies	Median threshold across studies: 2.40	OS: HR 2.180; RFS: HR 2.462	Elevated NLR was more frequent in higher-grade tumors and was associated with poorer OS and RFS.	Indirect; PNET-focused pooled evidence; interpretation is limited by differences between included studies
Lambrescu [46]	2022	Romania	Single-center retrospective with healthy controls	NENs of various primary sites	G1 5 G2 17 G3 3 Undetermined 6	N0 29 N1 12 M0 29 M1 12	31 NENs + 21 controls 10	Pretreatment assessment Surgery Endoscopic resection SSA Chemotherapy “Wait and see” policy	No prognostic cut-off; median NLR 3.01	Statistical significance reported; no HR	NLR, PLR, neutrophils, and SII were significantly higher in patients with NENs than in controls.	Indirect; mixed-primary study; no pancreatic-specific prognostic conclusion can be drawn
Casas [47]	2023	Spain Multicenter	Retrospective observational	GEP-NETs or NETs of unknown origin treated with PRRT	G1 17 G2 21 G3 2	Advanced GEP or unknown origin NETs	40 11	PRRT	2.61	PFS association: *p* = 0.001	Elevated baseline NLR was associated with significantly shorter PFS; post-treatment inflammatory indices were not prognostic.	Indirect; mixed-primary evidence in a specific treatment setting; pancreatic subgroup NR
Yang [48]	2024	China	Single-center retrospective	Surgically treated PNETs	G1 88 G2-3 70	I 64 II 70 III 5 IV 32	174 174	Preoperative assessment and curative resection	2.28	Univariate PFS: HR 2.352; not independent in multivariable analysis	High NLR was associated with larger tumors, higher grade, advanced TNM stage, and postoperative progression, but LMR—not NLR—remained independently prognostic.	Direct; pancreatic surgical cohort
Worapongpaibon [51]	2025	Meta-analysis	Systematic review and meta-analysis 105 studies	PDAC, PNET, and pancreatic cystic neoplasms; PNET subgroup analyzed	PNET-specific grade and differentiation distribution NR	Heterogeneous across included studies	20,138 NR	Mixed treatment settings	Meta-analysis subgroup threshold: 3.10	PNET OS: HR 2.57 multivariable; RFS: HR 3.05 multivariable	High NLR independently predicted worse OS and RFS in the PNET subgroup.	Indirect; relevant PNET subgroup evidence; Total meta-analysis population is not the PNET sample size
Wang [50]	2025	Meta-analysis	Meta-analysis 18 studies	GEP-NENs; PNEN subgroup analyzed	Grade and differentiation distribution NR	Mixed stages	2995 NR	Mixed treatment settings	Study-specific	PNEN OS: HR 3.98; RFS: HR 2.27	High NLR was associated with poorer OS and RFS in PNENs, particularly in surgically treated patients.	Indirect; relevant PNET subgroup evidence; Total meta-analysis population is not the PNET sample size
Ocakli [49]	2025	Turkey	Single-center retrospective	GEP-NENs	G1 64 G2 56 G3 11	Tumor size 1.7 (0.7–3.5) cm N0 99 N1 93	132 57	Surgery	Not specified	NLR: *p* = 0.998 for LN metastases	NLR was not predictive of lymph-node metastasis; tumor location, grade III, and tumor size ≥ 2 cm were independent predictors.	Indirect; mixed-primary evidence; the negative result cannot be assigned specifically to PNETs
Ye [53]	2025	China	Single-center retrospective; prognostic model development and validation	Surgically treated PNETs	G1 53 G2 40 G3 9	I-II 84 II-IV 18	102 102	Surgery; prognostic nomogram development	3.413	Independent in multivariate analysis for OS: HR 0.153	Low NLR was associated with shorter survival. NLR, PNI and AJCC stage integrated into prognostic nomogram for prediction of 3-, 5-, and 10-year OS	Direct; surgical PNET evidence

**Abbreviations:** AJCC, American Joint Committee on Cancer; AUC, area under the curve; DFS, disease-free survival; GE-NET, gastroenteric neuroendocrine tumor; GEP-NEN, gastroenteropancreatic neuroendocrine neoplasm; GEP-NET, gastroenteropancreatic neuroendocrine tumor; HR, hazard ratio; LN, lymph node; LMR, lymphocyte-to-monocyte ratio; NLR, neutrophil-to-lymphocyte ratio; NR, not reported in the reviewed section; OS, overall survival; PFS, progression-free survival; PLR, platelet-to-lymphocyte ratio; PNEN, pancreatic neuroendocrine neoplasm; PNET/pNET, pancreatic neuroendocrine tumor; PRRT, peptide receptor radionuclide therapy; RFS, recurrence-free survival; SSA, somatostatin analog. Table footnote/Legend: Green rows indicate studies with direct applicability to pancreatic neuroendocrine tumors (PanNETs), defined as homogeneous, surgical cohorts restricted entirely to pancreatic primaries. Yellow rows indicate studies with indirect applicability, including mixed cohorts encompassing multiple neuroendocrine neoplasm (NEN) primary sites, poorly differentiated carcinomas, or meta-analyses synthesizing heterogeneous constituent data.

**Table 3:** **Clinical utility of NLR.**

Utility	Rationale
**Preoperative risk stratification**	Associations between elevated NLR and higher tumor grade, advanced stage, metastatic disease, recurrence, and unfavorable survival outcomes
**Identifying patients at increased risk of recurrence**	Association between elevated preoperative NLR and shorter RFS, DFS and a higher risk of postoperative recurrence
**Postoperative surveillance**	Association between elevated preoperative NLR, particularly when additional high-risk features are present, and a higher risk of recurrence or disease progression
**Complementary biomarker**	Addition to established prognostic factors (Ki-67 proliferation index, WHO grade, TNM stage, lymph-node involvement, and distant metastasis) to prognostic scores and nomograms

Abbreviations: RFS, recurrence-free survival; DFS, disease-free survival; WHO, World Health Organization; TNM, Tumor, Node, Metastasis. Note: All listed utilities are currently investigational and represent research hypotheses rather than validated clinical strategies. Evidence regarding the prediction of surgical morbidity remains insufficient.

## Data Availability

No new data were created or analyzed in this study. Data sharing is not applicable to this article.
